# The longitudinal impact of the COVID-19 pandemic on health behaviors, psychosocial factors, and cognitive functioning in older adults

**DOI:** 10.3389/fnagi.2022.999107

**Published:** 2022-11-25

**Authors:** Hanna K. Hausman, Yunfeng Dai, Andrew O’Shea, Vanessa Dominguez, Matthew Fillingim, Kristin Calfee, Daniela Carballo, Cindy Hernandez, Sean Perryman, Jessica N. Kraft, Nicole D. Evangelista, Emily J. Van Etten, Samantha G. Smith, Pradyumna K. Bharadwaj, Hyun Song, Eric Porges, Steven T. DeKosky, Georg A. Hishaw, Michael Marsiske, Ronald Cohen, Gene E. Alexander, Samuel S. Wu, Adam J. Woods

**Affiliations:** ^1^Center for Cognitive Aging and Memory, McKnight Brain Institute, University of Florida, Gainesville, FL, United States; ^2^Department of Clinical and Health Psychology, College of Public Health and Health Professions, University of Florida, Gainesville, FL, United States; ^3^Department of Biostatistics, College of Public Health and Health Professions, College of Medicine, University of Florida, Gainesville, FL,, United States; ^4^Department of Neuroscience, College of Medicine, University of Florida, Gainesville, FL, United States; ^5^Brain Imaging, Behavior and Aging Laboratory, Department of Psychology and Evelyn F. McKnight Brain Institute, University of Arizona, Tucson, AZ, United States; ^6^Department of Neurology, College of Medicine, University of Florida, Gainesville, FL, United States; ^7^Department of Psychiatry, Neuroscience and Physiological Sciences Graduate Interdisciplinary Programs, and BIO5 Institute, University of Arizona and Arizona Alzheimer’s Disease Consortium, Tucson, AZ, United States

**Keywords:** COVID-19, older adults, health behaviors, psychosocial, cognition

## Abstract

**Background:** Older adults are at a greater risk for contracting and experiencing severe illness from COVID-19 and may be further affected by pandemic-related precautions (e.g., social distancing and isolation in quarantine). However, the longitudinal impact of the COVID-19 pandemic on older adults is unclear. The current study examines changes in health behaviors, psychosocial factors, and cognitive functioning in a large sample of older adults using a pre-pandemic baseline and longitudinal follow-up throughout 9 months of the COVID-19 pandemic.

**Methods:** One hundred and eighty-nine older adults (ages 65-89) were recruited from a multisite clinical trial to complete additional virtual assessments during the COVID-19 pandemic. Mixed effects models evaluated changes in health behaviors, psychosocial factors, and cognitive functioning during the pandemic compared to a pre-pandemic baseline and over the course of the pandemic (i.e., comparing the first and last COVID-19 timepoints).

**Results:** Compared to their pre-pandemic baseline, during the pandemic, older adults reported worsened sleep quality, perceived physical health and functioning, mental health, slight increases in depression and apathy symptoms, reduced social engagement/perceived social support, but demonstrated better performance on objective cognitive tasks of attention and working memory. Throughout the course of the pandemic, these older adults reported continued worsening of perceived physical health and function, fewer depression symptoms, and they demonstrated improved cognitive performance. It is important to note that changes on self-report mood measures and cognitive performance were relatively small regarding clinical significance. Education largely served as a protective factor, such that greater years of education was generally associated with better outcomes across domains.

**Conclusions:** The present study provides insights into the longitudinal impact of the COVID-19 pandemic on health behaviors, psychosocial factors, and cognitive functioning in a population disproportionately affected by the virus. Replicating this study design in a demographically representative older adult sample is warranted to further inform intervention strategies targeting older adults negatively impacted by the COVID-19 pandemic.

## Introduction

According to the CDC, older adults are at a greater risk for experiencing severe illness from COVID-19 that can result in hospitalization, intensive care, or even death (CDC, 2020). Adults 65 and older represent 16% of the United States population, yet this demographic accounts for ~75%–80% of COVID-19 deaths in the United States (Freed et al., 2020). As such, older adults are being encouraged to limit in-person interactions and abide by distancing and masking guidelines for an uncertain amount of time. While adhering to socialization restrictions may protect this population from contracting COVID-19, doing so may also threaten other aspects of well-being. Extensive research prior to COVID-19 has revealed negative effects of social isolation and perceived loneliness in older adults on mental health (Heikkinen and Kauppinen, 2004; Cornwell and Waite, 2009; Santini et al., 2020), physical health (Shankar et al., 2011; Cacioppo et al., 2014; Ong et al., 2016), and cognitive functioning (Tilvis et al., 2004; Wilson et al., 2007; Cacioppo and Hawkley, 2009; Shankar et al., 2013; Evans et al., 2019). However, in addition to social isolation, the COVID-19 pandemic presents stressors, including but not limited to, uncertainty due to inadequate information, health anxiety, limited access to healthcare and basic supplies, unmet personal needs, reduced social services, and job loss or income insecurity (Brooks et al., 2020; van Tilburg et al., 2020; Giebel et al., 2021).

To better understand the impact of COVID-19 on older adults, a systematic review by Lebrasseur et al. (2021) synthesized published results from early months of the COVID-19 pandemic (2019–October 2020). This review included 135 studies with participants 60 years of age and older. Of the 135 studies identified, only 11 were longitudinal studies that included a pre-pandemic timepoint (Hamm et al., 2020; Pierce et al., 2020; Shan Wong et al., 2020; Suzuki et al., 2020; van der Velden et al., 2020; van Tilburg et al., 2020; Whatley et al., 2020; Daly et al., 2021; Kivi et al., 2021; Krendl and Perry, 2021; Niedzwiedz et al., 2021). Overall, it was expected that the COVID-19 pandemic would negatively impact older adults’ engagement in health promoting behaviors (e.g., exercise, sleep, healthy diet) and mental health due to the implemented protective measures and resulting social isolation. Yet, this pattern of findings was not consistent across studies. For example, older adults reported increases in alcohol consumption and decreases in physical activity/increases in sedentary behaviors during the pandemic (Constant et al., 2020; Emerson, 2020; Malta et al., 2020; Suzuki et al., 2020; Niedzwiedz et al., 2021); however, some studies suggest that the magnitude of these negative health behavior changes during the pandemic is smaller for older adults compared to younger adults (Constant et al., 2020; Ferrante et al., 2020; Giustino et al., 2020; Malta et al., 2020; Rogers et al., 2020; Niedzwiedz et al., 2021). Similarly, Almondes et al. (2022) demonstrated negative changes in older adults’ reported sleep quality, whereas Pinto et al. (2020) suggested that older adults are less likely to complain about sleep difficulties during the early months of the pandemic. Furthermore, older adults in China reported engaging in adaptive health behaviors in response to the pandemic like eating a balanced diet, limiting alcohol, and maintaining adequate sleep schedules (Sun et al., 2020). The early literature regarding the pandemic’s impact on mental health in older adults reflects similar contradictions as the findings on health behaviors. For instance, some longitudinal studies showed no negative effect of the pandemic on depression, anxiety, or other mental health outcomes compared to a pre-pandemic timepoint in older adults (Hamm et al., 2020; Pierce et al., 2020; van der Velden et al., 2020; Daly et al., 2021; Kivi et al., 2021). In contrast, others have identified increases in depression, loneliness, anxiety, and insomnia in older adults during the initial months of the pandemic compared to a pre-pandemic baseline (Shan Wong et al., 2020; Krendl and Perry, 2021).

Since the Lebrasseur et al.’s (2021) systematic review, recent articles have also examined the impact of the COVID-19 pandemic on cognitive functioning in older adults. Cross sectional studies indicated no significant changes in subjective cognitive complaints in older adults during the first wave of the pandemic (De Pue et al., 2021; Fiorenzato et al., 2021). Longitudinal studies also did not indicate a change in objective cognitive performance on general cognition screeners compared to a pre-pandemic baseline (Amanzio et al., 2021). Further, throughout the early months of the pandemic, older adults’ performance remained stable on a working memory task but improved on a word recall task; however, the latter was no longer significant after the inclusion of mood measures in the model (Carbone et al., 2021). Overall, few studies have assessed longitudinal changes in objective measures of cognitive functioning in older adults both throughout the pandemic and compared to baseline functioning.

The heterogeneous findings regarding the pandemic’s impact on older adults may in part be related to differences in demographic and protective factors. For example, extensive literature prior to the COVID-19 pandemic suggests that education serves as a protective factor in late life, such that older adults with greater quantity or quality of education are less susceptible to age-related cognitive decline, brain changes, and dementia incidence (Qiu et al., 2001; Manly et al., 2005; Valenzuela and Sachdev, 2006; Stern, 2009; Teipel et al., 2009; Arenaza-Urquijo et al., 2013). Greater educational attainment has also been associated with better health literacy, physical functioning, mental health, and reduced mortality in older adults (Wolf et al., 2010; Louie and Ward, 2011; Patel et al., 2011; Chesser et al., 2016; Belo et al., 2020). Research grounded in the Fundamental Cause Theory (Link and Phelan, 1995) posits that education and health are interwoven given that educational attainment determines access to resources that promote health (i.e., income, healthcare, safe housing; Zajacova and Lawrence, 2018). Indeed, during the COVID-19 pandemic, greater educational attainment has been associated with decreased risk of contracting COVID-19 and reduced illness severity/hospitalizations (Chadeau-Hyam et al., 2020; Li et al., 2021; Yoshikawa and Asaba, 2021). As such, it would be important to explore the influence of educational attainment and other demographic factors in older adults’ ability to adapt and adjust to the pandemic over time.

Altogether, the impact of the COVID-19 pandemic on health behaviors, psychosocial factors, and cognitive functioning in older adults is unclear. It is important to note that several of the early studies did not include a pre-pandemic timepoint, making it difficult to establish a change from baseline functioning. Furthermore, longitudinal studies were largely conducted within the first few months of the COVID-19 pandemic and may only reflect unique short-term outcomes. At the time of this paper, the COVID-19 pandemic is still ongoing. Therefore, it is important to investigate the long-term effects of the COVID-19 pandemic in older adults with a prolonged exposure to pandemic-related events (i.e., changes in policies/procedures, waves of increased cases, vaccine distributions, etc.). The current study aims to fill the gaps in the literature by analyzing changes in health behaviors, psychosocial factors, and cognitive functioning in a sample of older adults using a pre-pandemic baseline and longitudinal follow-up throughout 9 months of the pandemic (dates ranging from April 2020 to August 2021). Compared to a pre-pandemic baseline, we hypothesized that our sample of healthy older adults will experience negative changes in health behaviors (Suzuki et al., 2020; Niedzwiedz et al., 2021) and psychosocial factors (Shan Wong et al., 2020; Krendl and Perry, 2021), but stable cognitive functioning during the pandemic (Amanzio et al., 2021; Carbone et al., 2021). Throughout 9 months of the pandemic, we hypothesized that our sample will experience improvements in health behaviors and psychosocial factors reflecting an overall adjustment to the times and continued stable cognitive functioning. Finally, we also explored the influence of demographic factors on health behaviors, psychosocial factors, and cognitive functioning in these longitudinal models.

## Materials and Methods

### Participants

Data were collected from participants that were enrolled in a multisite clinical trial: the Augmenting Cognitive Training in Older Adults study (ACT, R01AG054077^1^; Woods et al., 2018). Inclusion and exclusion criteria for the ACT study are detailed elsewhere (Woods et al., 2018). In brief, participants were between the ages of 65–89, had no history of major psychiatric illness [e.g., schizophrenia, substance dependence diagnosis, moderate to severe major depressive disorder (e.g., Beck Depression Inventory Scores greater than 20)], no history of brain or head injury resulting in loss of consciousness greater than 20 min, no unstable and chronic medical conditions (e.g., cancer, severe diabetes), and no formal diagnosis or evidence of mild cognitive impairment, dementia, or neurological brain disease. The Uniform Data Set (UDS) of the National Alzheimer’s Coordinating Center (NACC) was used to screen for individuals with possible mild cognitive impairment (MCI) or dementia (Weintraub et al., 2009). Possible MCI was defined by 1.5 standard deviations below the mean of age-, sex- and education-adjusted norms in any of the following domains: general cognition, memory, visuospatial, executive functioning/working memory, or language. Participants were also excluded if their total score on the Montreal Cognitive Assessment (MoCA)—a cognitive screening tool administered as part of the NACC UDS—was less than 20. All participants were right-handed and had no contraindications for neuroimaging protocols. Notably, in the parent trial, participants were randomized to receive an intervention that included a combination of transcranial direct current stimulation (active vs. sham) and cognitive training or educational training. Information regarding the randomization to different arms of the intervention cannot be accessed prior to completion of the parent trial analyses. As such, an assumption of randomized distribution of participants across intervention arms is being made for the present study. The total sample of the parent trial was 379 participants. The current study uses a subset of participants from the parent trial who signed an electronic consent form approved by the Institutional Review Board at the University of Florida and at the University of Arizona to participate in additional COVID-19 study procedures. The current sample demographic data is reported in Table 1, which included 189 healthy older adults ranging from 65 to 88 years old with 12–21 years of education recruited at the University of Florida and the University of Arizona. The racial demographics of the sample were as follows: 87.8% White, 4.8% Black or African American, 2.1% American Indian or Alaska Native, 2.1% identified as more than one race, 1.1% Asian, and 2.1% did not report. The sample also largely identified as not Hispanic or Latinx (92.6%).

**Table 1:** Sample demographics.

**Demographics (*n* = 189)**	**Mean (SD); *n* (%)**
Age	71.4 (4.8)
Sex (Number of Females)	123 (65.1%)
**Educational Attainment**	
Years of Education	16.5 (2.4)
GED or Equivalent	3 (1.6%)
High School Diploma	11 (5.8%)
Some College/Associate’s Degree	39 (20.6%)
Bachelor’s Degree	61 (32.3%)
Professional/Graduate Degree	75 (39.7%)
**Race**	
White	166 (87.8%)
Black or African American	9 (4.8%)
American Indian or Alaska Native	4 (2.1%)
Multiracial	4 (2.1%)
Asian American	2 (1.1%)
Not Reported	4 (2.1%)
**Ethnicity**	
Not Hispanic or Latinx	175 (92.6%)
Hispanic or Latinx	13 (6.9%)
Not Reported	1 (0.5%)

### Measures

For the parent trial, participants completed a comprehensive battery of assessments, questionnaires, and neuroimaging during an in-person screening visit and a baseline visit split over 2 days (~3 h per day). Notably, participants were not included in the parent trial if there was evidence of cognitive impairment (i.e., 1.5 standard deviations below normative data in a single domain on the NACC UDS or a MoCA score <20) or evidence of moderate or severe depression symptoms (i.e., Beck Depression Inventory score >20). Participants included in the present COVID-19 study completed their baseline visit prior to the issuance of stay-at-home orders for the COVID-19 pandemic (dates ranging from August 2017 to mid-March 2020). These participants then completed an assessment visit during the early months of the COVID-19 pandemic (i.e., COVID-19 T1) with dates ranging from April 2020 to December 2020. Follow-up assessments were completed monthly for 6 months (COVID-19 T2–7), and a final assessment (COVID-19 T8) was completed 9 months from COVID-19 Timepoint 1 (dates ranging from January 2021 to August 2021). COVID-19 T8 was not considered a “post-COVID-19” timepoint but rather the final “during COVID-19” assessment, as it was collected while the pandemic was ongoing along with COVID-19 T1–7. To minimize risk of exposure and abide by social distancing restrictions, the questionnaires and assessments given during COVID-19 were adapted for remote administration. An abbreviated version of the Montreal Cognitive Assessment described below (Wittich et al., 2010; Pendlebury et al., 2013; Katz et al., 2021) and the NACC UDS Number Span Test were administered *via* telephone, while the remaining questionnaires were completed through a secure web platform (REDCap) *via* computer or tablet. Participants without access to devices or internet had the option to participate solely *via* telephone. For the subset of measures described below, the corresponding in-person Montreal Cognitive Assessment and Number Span Test as part of the NACC UDS were completed at the screening visit, and the remaining measures were completed across the two baseline visits. Measures are divided into domains assessing health behaviors, psychosocial factors, and cognitive functioning. Summary statistics of the measures and sample sizes at each timepoint are provided in Table 2.

**Table 2:** Assessments by timepoint.

**Variable**	**Value**	**Pre-Pandemic Baseline (*N* = 189)**	**COVID-19 T1 (*N* = 189)**	**COVID-19 T2 (*N* = 176)**	**COVID-19 T3 (*N* = 178)**	**COVID-19 T4 (*N* = 170)**	**COVID-19 T5 (*N* = 171)**	**COVID-19 T6 (*N* = 168)**	**COVID-19 T7 (*N* = 157)**	**COVID-19 T8 (*N* = 156)**
**Health Behavior Measures**										
AUDIT	Mean ± SD	2.5 ± 2.4	2.4 ± 2.2	2.3 ± 2.2	2.3 ± 2.3	2.3 ± 2.1	2.4 ± 2.2	2.4 ± 2.4	2.5 ± 2.4	2.3 ± 2.4
	Median (min, max)	2 (0, 18)	2 (0, 13)	2 (0, 14)	2 (0, 16)	2 (0, 14)	2 (0, 13)	2 (0, 13)	2 (0, 13)	2 (0, 15)
	Missing	0	2 (1.1%)	4 (2.3%)	5 (2.8%)	2 (1.2%)	7 (4.1%)	7 (4.2%)	5 (3.2%)	8 (5.1%)
PSQI	Mean ± SD	5.5 ± 3.2	6.2 ± 3.4	6.2 ± 3.7	6.0 ± 3.5	5.9 ± 3.3	6.0 ± 3.4	6.1 ± 3.5	5.9 ± 3.3	6.1 ± 3.5
	Median (min, max)	5 (0, 16)	6 (1, 17)	6 (1, 17)	5 (1, 17)	5 (1, 16)	6 (1, 15)	6 (0, 16)	5 (1, 16)	6 (1, 17)
	Missing	2 (1.1%)	4 (2.1%)	9 (5.1%)	8 (4.5%)	5 (2.9%)	8 (4.7%)	10 (6.0%)	7 (4.5%)	10 (6.4%)
SF-36 Physical Health	Mean ± SD	80.0 ± 17.0	75.2 ± 19.7	76.3 ± 17.9	76.0 ± 18.6	75.8 ± 18.6	75.6 ± 19.4	74.2 ± 20.0	75.1 ± 19.3	74.0 ± 18.3
	Median (min, max)	86 (18, 100)	81 (19, 100)	81 (18, 100)	80 (20, 100)	81 (10, 100)	80 (16, 100)	78 (10, 100)	79 (11, 100)	78 (13, 100)
	Missing	0	2 (1.1%)	4 (2.3%)	5 (2.8%)	2 (1.2%)	7 (4.1%)	7 (4.2%)	5 (3.2%)	8 (5.1%)
PROMIS Physical Function	Mean ± SD	36.4 ± 5.6	35.5 ± 5.7	36.0 ± 5.4	35.4 ± 5.6	35.1 ± 6.0	35.1 ± 6.1	35.5 ± 5.9	35.1 ± 6.3	35.1 ± 5.9
	Median (min, max)	38 (8, 40)	38 (13, 40)	38 (9, 40)	37 (8, 40)	37 (10, 40)	38 (8, 40)	38 (13, 40)	38 (8, 40)	37 (11, 40)
	Missing	0	1 (0.5%)	4 (2.3%)	5 (2.8%)	2 (1.2%)	7 (4.1%)	7 (4.2%)	4 (2.5%)	8 (5.1%)
**Psychosocial Measures**										
SF-36 Mental Health	Mean ± SD	81.6 ± 14.1	73.7 ± 18.2	74.7 ± 17.9	75.1 ± 17.6	75.4 ± 18.0	75.8 ± 19.2	76.5 ± 17.5	76.0 ± 18.7	74.4 ± 18.3
	Median (min, max)	86 (35, 100)	80 (27, 100)	78 (27, 100)	80 (6, 100)	81 (27, 100)	84 (24, 100)	80 (27, 100)	82 (26, 100)	80 (22, 100)
	Missing	0	2 (1.1%)	4 (2.3%)	5 (2.8%)	2 (1.2%)	7 (4.1%)	7 (4.2%)	5 (3.2%)	8 (5.1%)
BDI-II	Mean ± SD	3.4 ± 3.9	6.9 ± 6.4	6.4 ± 6.1	6.2 ± 5.9	5.7 ± 5.9	5.9 ± 6.3	6.0 ± 6.1	5.8 ± 6.0	6.0 ± 6.0
	Median (min, max)	2 (0, 18)	5 (0, 33)	5 (0, 27)	4 (0, 28)	4 (0, 30)	4 (0, 28)	4 (0, 28)	5 (0, 28)	5 (0, 29)
	Missing	0	1 (0.5%)	4 (2.3%)	5 (2.8%)	2 (1.2%)	7 (4.1%)	7 (4.2%)	4 (2.5%)	8 (5.1%)
	Missing (Imputed)	0	1 (0.5%)	0	0	0	0	0	0	0
STAI State Anxiety	Mean ± SD	28.4 ± 8.3	29.5 ± 9.4	29.1 ± 9.2	28.6 ± 9.4	28.0 ± 9.2	27.9 ± 9.6	28.2 ± 9.9	28.7 ± 9.8	28.9 ± 10.0
	Median (min, max)	26 (20, 58)	26 (20, 60)	26 (20, 60)	25 (20, 61)	25 (20, 64)	24 (20, 56)	24 (20, 55)	24 (20, 60)	26 (20, 62)
	Missing	0	1 (0.5%)	4 (2.3%)	5 (2.8%)	2 (1.2%)	7 (4.1%)	7 (4.2%)	4 (2.5%)	8 (5.1%)
	Missing (Imputed)	0	1 (0.5%)	0	0	0	0	0	0	0
STAI Trait Anxiety	Mean ± SD	28.6 ± 7.4	29.5 ± 9.2	29.3 ± 9.0	28.3 ± 8.8	27.9 ± 9.0	28.0 ± 9.4	28.3 ± 9.4	28.6 ± 9.7	28.4 ± 9.1
	Median (min, max)	26 (20, 56)	26 (20, 64)	26 (20, 62)	25 (20, 60)	25 (20, 65)	25 (20, 64)	24.5 (20, 67)	25 (20, 66)	25 (20, 61)
	Missing	0	1 (0.5%)	4 (2.3%)	5 (2.8%)	2 (1.2%)	7 (4.1%)	7 (4.2%)	4 (2.5%)	8 (5.1%)
	Missing (Imputed)	0	1 (0.5%)	0	0	0	0	0	0	0
Apathy Scale	Mean ± SD	9.1 ± 4.7	11.4 ± 5.3	11.4 ± 5.2	11.3 ± 5.4	10.8 ± 5.7	11.0 ± 5.7	11.0 ± 5.1	11.3 ± 5.7	11.4 ± 5.8
	Median (min, max)	8 (1, 26)	11 (2, 26)	10 (2, 27)	11 (2, 25)	10 (1, 30)	10 (2, 31)	10 (2, 27)	10 (2, 28)	11 (0, 31)
	Missing	0	1 (0.5%)	4 (2.3%)	5 (2.8%)	2 (1.2%)	7 (4.1%)	7 (4.2%)	4 (2.5%)	8 (5.1%)
	Missing (Imputed)	0	1 (0.5%)	4 (2.3%)	5 (2.8%)	2 (1.2%)	7 (4.1%)	7 (4.2%)	4 (2.5%)	8 (5.1%)
UCLA Loneliness Scale	Mean ± SD	34.7 ± 8.7	36.2 ± 10.4	36.1 ± 10.5	35.6 ± 10.8	35.5 ± 10.8	34.8 ± 10.6	34.7 ± 10.9	34.7 ± 11.6	34.9 ± 11.3
	Median (min, max)	34 (20, 59)	34 (20, 65)	35 (20, 69)	34 (20, 69)	33 (20, 69)	32 (20, 65)	32 (20, 73)	32 (20, 76)	32 (20, 71)
	Missing	0	1 (0.5%)	4 (2.3%)	5 (2.8%)	2 (1.2%)	7 (4.1%)	7 (4.2%)	3 (1.9%)	9 (5.8%)
Lubben Social Network Scale	Mean ± SD	38.6 ± 8.0	33.9 ± 8.8	34.0 ± 9.0	34.0 ± 9.1	34.1 ± 9.3	34.1 ± 9.0	34.4 ± 8.4	33.9 ± 8.9	34.4 ± 8.4
	Median (min, max)	40 (2, 59)	35 (4, 50)	36 (6, 51)	35 (0, 52)	35 (0, 51)	35.5 (4, 52)	36 (0, 52)	36 (4, 49)	35 (11, 51)
	Missing	0	1 (0.5%)	4 (2.3%)	5 (2.8%)	2 (1.2%)	7 (4.1%)	7 (4.2%)	4 (2.5%)	8 (5.1%)
**Cognition**										
MoCA 22-point	Mean ± SD	19.7 ± 1.6	19.2 ± 2.0	19.5 ± 1.9	19.5 ± 2.1	19.7 ± 1.8	19.9 ± 1.9	20.1 ± 1.9	20.1 ± 1.5	20.2 ± 1.6
	Median (min, max)	20 (15, 22)	19 (11, 22)	20 (13, 22)	20 (10, 22)	20 (11, 22)	20 (11, 22)	21 (12, 22)	20 (16, 22)	21 (13, 22)
	Missing	0	1 (0.5%)	0	4 (2.2%)	1 (0.6%)	4 (2.3%)	5 (3.0%)	3 (1.9%)	7 (4.5%)
PROMIS Applied Cognitive Abilities	Mean ± SD	32.1 ± 6.4	31.0 ± 6.4	31.6 ± 6.2	31.4 ± 6.4	32.0 ± 6.2	32.0 ± 6.3	31.3 ± 6.7	31.5 ± 6.3	31.3 ± 6.7
	Median (min, max)	33 (8, 40)	32 (10, 40)	32 (9, 40)	32 (10, 40)	33 (9, 40)	32 (12, 40)	32 (10, 40)	32 (13, 40)	32 (11, 40)
	Missing	0	1 (0.5%)	4 (2.3%)	5 (2.8%)	2 (1.2%)	7 (4.1%)	7 (4.2%)	4 (2.5%)	8 (5.1%)
NACC UDS Number Span Forward	Mean ± SD	8.2 ± 2.0	8.7 ± 2.3	8.8 ± 2.2	9.0 ± 2.2	8.9 ± 2.2	9.3 ± 2.4	9.5 ± 2.3	9.4 ± 2.2	9.4 ± 2.3
	Median (min, max)	8 (4, 13)	9 (4, 14)	9 (1, 14)	9 (3, 14)	9 (3, 14)	9 (3, 14)	9 (4, 14)	9 (5, 14)	9 (4, 14)
	Missing	0	1 (0.5%)	0	4 (2.2%)	2 (1.2%)	4 (2.3%)	5 (3.0%)	3 (1.9%)	8 (5.1%)
NACC UDS Number Span Backward	Mean ± SD	7.0 ± 2.0	7.9 ± 2.3	8.0 ± 2.3	8.1 ± 2.3	8.4 ± 2.3	8.7 ± 2.5	8.5 ± 2.5	8.6 ± 2.4	8.6 ± 2.6
	Median (min, max)	7 (3, 13)	8 (0, 14)	8 (2, 14)	8 (0, 14)	8 (0, 14)	9 (0, 14)	8 (2, 14)	8 (3, 14)	8 (2, 14)
	Missing	0	1 (0.5%)	0	4 (2.2%)	2 (1.2%)	4 (2.3%)	5 (3.0%)	3 (1.9%)	8 (5.1%)
Assessment Date	Median (min, max)	01/24/19 (08/21/17, 03/11/20)	05/20/20 (04/30/20, 12/10/20)	06/22/20 (06/01/20, 01/13/21)	07/21/20 (06/30/20, 02/13/21)	08/27/20 (07/30/20, 03/19/21)	09/29/20 (08/31/20, 04/20/21)	11/01/20 (09/29/20, 05/10/21)	12/08/20 (11/02/20, 06/10/21)	02/08/21 (01/04/21, 08/10/21)

Notes: AUDIT, Alcohol Use Disorders Identification Test; PSQI, Pittsburgh Sleep Quality Index; SF-36, Short Form 36 Health Survey Questionnaire; PROMIS, Patient-Reported Outcomes Measurement Information System; BDI-II, Beck Depression Inventory—Second Edition; STAI, State-Trait Anxiety Inventory; UCLA, University of California, Los Angeles; MoCA 22-point, Montreal Cognitive Assessment abbreviated version based on 22 points; NACC UDS, National Alzheimer’s Coordinating Center Uniform Data Set. The rows of missing values are the number/percentage of participants without total scores for the measure at each timepoint. For the BDI-II, STAI State and Trait Anxiety, and Apathy Scale, the table includes a row for number/percentage of participants with missing total scores following imputation procedures for missing items.

### Measures of health behaviors

#### Alcohol Use Disorders Identification Test (AUDIT)

The Alcohol Use Disorders Identification Test (AUDIT) is an alcohol use screening instrument developed by the World Health Organization (Saunders et al., 1993). It consists of 10 items assessing alcohol consumption (e.g., “How often do you have a drink containing alcohol?”), drinking behaviors (e.g., “How often during the last year have you found that you were not able to stop drinking once you had started?”), and alcohol-related problems (e.g., “Have you or someone else been injured as a result of your drinking?”). Total scores range from 0 to 40 where 0 represents an abstainer from alcohol and greater scores represent higher risk alcohol use/dependence.

#### Pittsburgh Sleep Quality Index (PSQI)

The Pittsburgh Sleep Quality Index (PSQI) is a self-report measure of sleep quality and sleep disturbances over a 1-month interval (Buysse et al., 1989). Individual items create seven component scores for sleep quality, sleep latency, sleep duration, sleep efficiency, sleep disturbance, use of sleep medication, and daytime dysfunction. The sum of component scores yields a global score, and global score of 5 or greater is indicative of poor sleep quality.

#### Short Form 36 Health Survey Questionnaire (SF-36)—Physical Health

The Short Form 36 Health Survey Questionnaire (SF-36) is a measure consisting of 36 items that provides eight health domain scores, which are combined to create two component scores: the physical health summary and the mental health summary (Ware and Sherbourne, 1992). The physical health summary consists of the physical functioning (10 items), physical role limitations (four items), bodily pain (two items), and general health perception scales (five items). Greater scores represent better physical health.

#### Patient-Reported Outcomes Measurement Information System (PROMIS)—Physical Function Short Form 8b

Patient-Reported Outcomes Measurement Information System (PROMIS) is a set of person-centered measures funded by the National Institute on Health that assesses physical, mental, and social health outcomes in adults and children (Cella et al., 2010; Hays et al., 2018). The PROMIS physical scale consists of eight items assessing one’s ability to complete daily tasks and physical limitations. Items 1–4 ask participants to rate their ability to complete tasks like household chores, walking up and down stairs, walking for 15 min, and shopping from “without any difficulty” to “unable to do.” Items 5–8 ask participants to rate how much their health limits their physical ability to do 2 h of labor, moderate housework (e.g., vacuuming, sweeping), carrying groceries, and heavy housework (e.g., scrubbing floors, moving furniture) from “not at all” to “cannot do.” Greater scores represent better physical functioning.

### Measures of psychosocial factors

#### Short Form 36 Health Survey Questionnaire (SF-36)—Mental Health

The SF-36 mental health summary consists of the vitality (four items), social functioning (two items), emotional role limitations (three items), and mental health scales (five items; Ware et al., 1994). Greater scores represent better mental health.

#### Beck Depression Inventory—Second Edition (BDI-II)

The Beck Depression Inventory—second edition (BDI-II) is a self-report questionnaire with 21 items evaluating depression symptoms over the last 2 weeks (e.g., low mood, pessimism, sense of failure, self-dissatisfaction, etc.; Beck et al., 1961). Recommended cut-off scores for total scores are as follows: 0–13 minimal, 14–19, mild, 20–28 moderate, and 29–63 severe symptoms.

#### State Trait Anxiety Inventory (STAI)

The State Trait Anxiety Inventory (STAI) is a self-report questionnaire with 40 self-descriptive statements (e.g., I feel calm). The participant uses a 4-point Likert scale ranging from “not at all” to “very much so” to rate the first 20 items indicating how they currently feel (i.e., state anxiety). Then, the participant uses a 4-point Likert scale ranging from “almost never” to “almost always” to rate the remaining 20 items indicating how they generally feel (i.e., trait anxiety; Spielberger et al., 1983). Scores range from 20 to 80, and higher scores represent greater levels of anxiety. A cut-off score of 39–40 has been suggested for clinically significant levels of anxiety (Julian, 2011).

#### Starkstein Apathy Scale

The Apathy Scale is a 14-item self-report questionnaire (Starkstein et al., 1992). The participant answers questions about behavioral, cognitive, and emotional indicators of apathy (e.g., “Are you interested in learning new things?,” “Do you have motivation?,” “Are you neither happy nor sad, just in between?”) using a 4-point Likert scale ranging from “not at all” to “a lot.” A cut-off score of 14 has been suggested for clinically significant levels of apathy.

#### University of California, Los Angeles (UCLA) Loneliness Scale

The University of California, Los Angeles (UCLA) Loneliness Scale is a 20-item self-report questionnaire used to measure subjective feelings of loneliness and social isolation (Russell et al., 1978). Participants rate items (e.g., “How often do you feel alone?”) using a 4-point Likert scale ranging from “never” to “often.” Higher scores represent more loneliness.

#### Lubben Social Network Scale—Revised (SNS-R)

The Social Network Scale—Revised (SNS-R) is a 12-item self-report questionnaire used to measure perceived social support received by friends and family and to gauge social isolation in older adults (Lubben, 1988). The items measure the size, closeness, and contact frequency of a participant’s social network (e.g., “How many relatives do you feel close to such that you would call on them for help?”). Total scores range from 0 to 60 with higher scores representing greater social engagement/perceived social support and lower risk for social isolation.

### Measures of cognitive functioning

#### Montreal Cognitive Assessment (MoCA)

The Montreal Cognitive Assessment (MoCA) is a cognitive screening tool used to detect global cognitive functioning in domains of attention, concentration, executive functions, memory, language, visuospatial skills, abstraction, calculation, and orientation (Nasreddine et al., 2005). The full version of the MoCA was administered at the screening in-person assessment. Total scores range from 0 to 30 with a traditional cutoff score of 26 to differentiate cognitive impairment from normal. However, optimal cutoff scores for detection of mild cognitive impairment and dementia differ by racial/ethnic group and years of education (Milani et al., 2018). An abbreviated version of the measure was administered *via* telephone on the assessments during the pandemic. The abbreviated version removes the visuospatial elements and is scored out of 22 points with a suggested cut-off score of 18 (Wittich et al., 2010; Pendlebury et al., 2013). To our knowledge, there are no demographically adjusted cut-off scores for this abbreviated version of the MoCA.

#### Patient-Reported Outcomes Measurement Information System (PROMIS)—Applied Cognitive Abilities Short Form 8a

The PROMIS cognitive abilities scale consists of eight items assessing one’s perceived cognitive functioning (e.g., “My mind has been as sharp as usual.” “I have been able to think clearly without extra effort.”; Saffer et al., 2014). Participants are asked to rate the items using a 5-point Likert Scale from “not at all” to “very much” over the past 7 days. Greater scores represent better cognitive functioning.

#### National Alzheimer’s Coordinating Center Uniform Data Set (NACC UDS) Number Span Test—Forward and Backward

The Number Span Forward Test is an auditory attention task (Weintraub et al., 2018). Participants read a sequence of numbers and must recall the numbers in the same order, increasing in sequence length with correct trials. The Number Span Backward Test is a verbal working memory task. Participants read a sequence of numbers and must recall the numbers in backwards order from the presentation (e.g., “1, 2, 3” = “3, 2, 1”). For both tasks, higher scores represent better task performance. Although the MoCA contains a task analogous to the Number Span Test, this test was included in the present study to more comprehensively measure attention and working memory, as it contains several more trials than the MoCA, and to comment on changes specific to attention and working memory since the MoCA produces a total score including performance across additional domains (e.g., memory, naming, language, etc.).

### Statistical analyses

#### Data preparation

There were no missing items on assessments administered over the phone during the COVID-19 assessments (i.e., MoCA and Number Span Test). However, there were missing items on questionnaires that participants completed online *via* RedCap. For psychometric scales with clinical cut-off scores (i.e., BDI-II, STAI, Apathy Scale), we used a two-step strategy to impute missing data. First, for all items with the same range of score, if the percent of missing items was less than 25% of total items in a questionnaire or assessment, we estimated the total score with the following formula:


$$Total\;score=\frac{Total\;score\;for\;nonmissing\;items\;X\;Total\;number\;of\;items}{Number\;of\;nonmissing\;items}$$

Second, if the percent of missing items was greater than 25%, we used the method of “last observation carried forward” to impute the scores, where a participant’s total score is carried over from the previous timepoint (Siddiqui, 2015). The SF-36 measures are scored using an average of all non-missing items. Total scores for the UCLA Loneliness Scale, SNS-R, and AUDIT were calculated ignoring missing items. If all items of a questionnaire or assessment were missing, we considered the total score of the questionnaire or assessment as missing for that timepoint (Table 2).

Extreme outliers were identified as values that fall outside of three times the interquartile range (IQR) for each measure across all timepoints. There was a total of 39 cases within 16 participants that met this criterion (BDI-II = 2, STAI State Anxiety = 1, PROMIS Physical Function = 12, AUDIT = 13, MoCA = 11); however, 36 of these cases were within individuals who flagged as either a minor (beyond 1.5 times the IQR) or extreme (beyond three times the IQR) outlier at multiple timepoints. For example, on eight out of their nine timepoints, one participant was identified as an extreme outlier on the AUDIT measure, indicative of consistent reporting of high alcohol use within the individual over time. As such, data were considered to be informative and accurate reporting of functioning, and no cases were excluded in the following analyses.

#### Statistical models

Mixed effects models were used to calculate fixed effects and evaluate change in health behaviors, psychosocial factors, and cognitive functioning over the period of this study controlling for age, sex, race, ethnicity, highest academic degree, and clinical trial site. Given the unbalanced distribution of participants represented across racial categories, the variable for race was collapsed across two groups—those that identify as White and those that identify with a race group other than White. While collapsing this variable preserves model fit, it hinders the ability to examine differences across racial groups, particularly those at greater risk for contracting COVID-19 (American Indian or Alaska Native, Black or African American, and Hispanic or Latinx individuals; CDC, 2022a) and is ultimately a limitation of the study that would be important to evaluate in future work. Additionally, for educational attainment, those who earned a high school diploma or a high school equivalency degree through passing the General Educational Development tests (i.e., obtaining a GED) were combined into the same level due to few participants in each. Change in outcomes were evaluated through two main contrasts. First, participants’ pre-pandemic baseline outcomes were compared to an average of all COVID-19 timepoints to emulate *during* pandemic functioning (i.e., Pre-Pandemic Baseline vs. Average COVID-19 T1–T8). Second, change in outcomes *throughout* the pandemic were evaluated by comparing participants outcomes between the first and last COVID-19 timepoint (i.e., COVID-19 T1 vs. COVID-19 T8). Restricted maximum likelihood and an unstructured covariance matrix were used to estimate the model. The significant level was set as 0.05. All analyses were carried out by using software SAS v9.4 (SAS Institute Inc., Cary, NC, USA) and R v3.5.2 (The R Foundation for Statistical Computing Platform; Tables s 3–5).

**Table 3:** Health behavior results.

**Effect**	**Variable Category**	**AUDIT**		**PSQI**		**SF-36 Physical**		**PROMIS Physical**	
		**Estimate**	***P*-Value**	**Estimate**	***P*-Value**	**Estimate**	***P*-Value**	**Estimate**	***P*-Value**
Intercept		0.08	0.97	−0.31	0.93	112.95	0.00	45.34	<0.001
Age		0.02	0.46	0.07	0.12	−0.56	0.02	−0.15	0.05
Sex	Female	−0.60	0.05	1.16	0.01	−4.66	0.05	−2.04	0.01
	Male	0.00		0.00		0.00		0.00	
Race	Other Racial Groups	−0.98	0.04	−1.02	0.15	−1.18	0.75	0.66	0.57
	White	0.00		0.00		0.00		0.00	
Ethnicity	Hispanic or Latinx	0.27	0.66	1.16	0.22	−2.88	0.55	−0.15	0.92
	Not Hispanic or Latinx	0.00		0.00		0.00		0.00	
Highest Academic Degree	Some college/Associate’s Degree	1.23	0.05	−0.13	0.89	12.45	0.01	3.83	0.01
	Bachelor’s Degree	1.19	0.05	−0.64	0.46	16.69	<0.001	4.69	0.001
	Professional/Graduate Degree	1.99	<0.001	−0.61	0.48	15.04	0.001	4.81	<0.001
	High School/GED	0.00		0.00		0.00		0.00	
Site	University of Florida	−0.30	0.33	0.75	0.10	−6.67	0.01	−2.02	0.01
	University of Arizona	0.00		0.00		0.00		0.00	
Timepoint	COVID-19 T1	−0.10	0.27	0.72	<0.001	−4.98	<0.001	−0.88	<0.01
	COVID-19 T2	−0.23	0.02	0.78	<0.001	−4.94	<0.001	−0.76	0.01
	COVID-19 T3	−0.15	0.13	0.43	0.01	−4.63	<0.001	−1.09	<0.001
	COVID-19 T4	−0.17	0.11	0.45	0.01	−4.38	<0.001	−1.22	<0.001
	COVID-19 T5	−0.18	0.06	0.51	0.01	−4.68	<0.001	−1.26	<0.001
	COVID-19 T6	−0.13	0.24	0.77	<0.001	−6.33	<0.001	−0.99	<0.01
	COVID-19 T7	−0.05	0.66	0.61	<0.001	−5.92	<0.001	−1.56	<0.001
	COVID-19 T8	−0.20	0.05	0.76	<0.001	−7.17	<0.001	−1.70	<0.001
	Pre-Pandemic Baseline	0.00		0.00		0.00		0.00	
Contrast	Pre-Pandemic Baseline vs. COVID-19 T1–T8	−0.15	0.08	0.63	<0.001	−5.38	<0.001	−1.18	<0.001
	COVID-19 T1 vs. COVID-19 T8	−0.10	0.20	0.04	0.82	−2.19	0.02	−0.83	0.01

Notes: AUDIT, Alcohol Use Disorders Identification Test; PSQI, Pittsburgh Sleep Quality Index; SF-36 Physical, Short Form 36 Health Survey Questionnaire Physical Health Summary; PROMIS Physical, Patient-Reported Outcomes Measurement Information System Physical Function. This table contains the model estimates and respective significance values for the main effects of each covariate and timepoint, as well as, the contrasts of interest. The contrast “Pre-Pandemic Baseline vs. COVID-19 T1–T8” compares each measure obtained at a baseline assessment prior to the pandemic to an average of the same measure collected across eight assessments during the pandemic (i.e., the average of COVID-19 timepoints 1 through 8). The contrast “COVID-19 T1 vs. COVID-19 T8” compares each measure obtained at the first assessment during the pandemic compared to the last assessment during the pandemic (i.e., COVID-19 timepoint 1 vs. COVID-19 timepoint 8).

**Table 4:** Psychosocial factors results.

**Effect**	**Variable Category**	**SF-36 Mental**		**BDI-II**		**STAI—State**		**STAI—Trait**		**Apathy Scale**		**UCLA Loneliness**		**Lubben Social Network Scale**	
		**Estimate**	***P*-Value**	**Estimate**	***P*-Value**	**Estimate**	***P*-Value**	**Estimate**	***P*-Value**	**Estimate**	***P*-Value**	**Estimate**	***P*-Value**	**Estimate**	***P*-Value**
Intercept		86.81	<0.001	−5.46	0.21	20.16	0.01	31.58	0.00	6.78	0.17	36.01	<0.001	53.68	<0.001
Age		−0.23	0.25	0.15	0.01	0.15	0.16	0.02	0.85	0.07	0.32	0.07	0.61	−0.27	0.02
Sex	Female	−3.50	0.08	0.49	0.41	1.24	0.23	1.03	0.33	−0.22	0.74	−0.88	0.50	0.91	0.43
	Male	0.00		0.00		0.00		0.00		0.00		0.00		0.00	
Race	Other Racial Groups	2.30	0.46	0.80	0.38	0.81	0.61	2.10	0.19	−0.46	0.65	1.26	0.53	−0.86	0.63
	White	0.00		0.00		0.00		0.00		0.00		0.00		0.00	
Ethnicity	Hispanic or Latinx	−5.07	0.22	−0.45	0.71	−0.17	0.93	−2.54	0.23	−0.23	0.86	0.09	0.97	−2.41	0.31
	Not Hispanic or Latinx	0.00		0.00		0.00		0.00		0.00		0.00		0.00	
Highest Academic Degree	Some College /Associate’s Degree	14.22	<0.001	−1.52	0.19	−3.35	0.11	−4.79	0.02	−2.85	0.03	−6.28	0.02	6.67	<0.01
	Bachelor’s Degree	15.71	<0.001	−2.50	0.03	−2.88	0.15	−3.53	0.08	−2.17	0.08	−5.13	0.04	3.13	0.16
	Professional /Graduate Degree	15.07	<0.001	−2.23	0.04	−3.35	0.09	−5.52	0.01	−3.00	0.01	−6.50	0.01	4.81	0.03
	High School /GED	0.00		0.00		0.00		0.00		0.00		0.00		0.00	
Site	University of Florida	−0.45	0.82	−0.61	0.29	−0.53	0.61	−1.44	0.17	0.48	0.46	−0.34	0.79	−1.17	0.31
	University of Arizona	0.00		0.00		0.00		0.00		0.00		0.00		0.00	
Timepoint	COVID-19 T1	−8.21	<0.001	3.52	<0.001	1.12	0.16	0.94	0.12	2.30	<0.001	1.54	0.01	−4.61	<0.001
	COVID-19 T2	−7.36	<0.001	3.03	<0.001	0.81	0.27	0.56	0.34	2.28	<0.001	1.27	0.03	−4.70	<0.001
	COVID-19 T3	−6.95	<0.001	2.77	<0.001	0.28	0.73	−0.21	0.71	2.21	<0.001	0.93	0.12	−4.73	<0.001
	COVID-19 T4	−6.48	<0.001	2.32	<0.001	−0.25	0.76	−0.59	0.32	1.87	<0.001	0.98	0.12	−4.57	<0.001
	COVID-19 T5	−6.30	<0.001	2.46	<0.001	−0.42	0.61	−0.51	0.40	2.00	<0.001	0.50	0.43	−4.88	<0.001
	COVID-19 T6	−6.16	<0.001	2.72	<0.001	0.13	0.88	−0.06	0.92	2.06	<0.001	0.64	0.31	−4.64	<0.001
	COVID-19 T7	−6.51	<0.001	2.59	<0.001	0.70	0.40	0.17	0.78	2.27	<0.001	0.74	0.26	−4.93	<0.001
	COVID-19 T8	−8.06	0.00	2.63	<0.001	0.93	0.31	0.08	0.91	2.55	<0.001	1.03	0.14	−5.00	<0.001
	Pre-Pandemic Baseline	0.00		0.00		0.00		0.00		0.00		0.00		0.00	
Contrast	Pre-Pandemic Baseline vs. COVID-19 T1–T8	−7.00	<0.001	2.75	<0.001	0.41	0.58	0.05	0.93	2.19	<0.001	0.95	0.09	−4.76	<0.001
	COVID-19 T1 vs. COVID-19 T8	0.16	0.90	−0.89	0.04	−0.19	0.77	−0.86	0.06	0.25	0.48	−0.52	0.36	−0.39	0.34

Notes: SF-36 Mental, Short Form 36 Health Survey Questionnaire Mental Health Summary; BDI-II, Beck Depression Inventory—Second Edition; STAI, State-Trait Anxiety Inventory; UCLA, University of California, Los Angeles. This table contains the model estimates and respective significance values for the main effects of each covariate and timepoint, as well as, the contrasts of interest. The contrast “Pre-Pandemic Baseline vs. COVID-19 T1–T8” compares each measure obtained at a baseline assessment prior to the pandemic to an average of the same measure collected across eight assessments during the pandemic (i.e., the average of COVID-19 timepoints 1 through 8). The contrast “COVID-19 T1 vs. COVID-19 T8” compares each measure obtained at the first assessment during the pandemic compared to the last assessment during the pandemic (i.e., COVID-19 timepoint 1 vs. COVID-19 timepoint 8).

**Table 5:** Cognitive functioning results.

**Effect**	**Variable Category**	**MoCA 22-Point**		**PROMIS Applied Cognitive Abilities**		**NACC UDS Number Span Forward**		**NACC UDS Number Span Backward**	
		**Estimate**	***P*-Value**	**Estimate**	***P*-Value**	**Estimate**	***P*-Value**	**Estimate**	***P*-Value**
Intercept		23.65	<0.001	33.35	<0.001	10.78	<0.001	8.21	<0.001
Age		−0.07	<0.001	−0.06	0.44	−0.05	0.04	−0.03	0.25
Sex	Female	0.23	0.17	−1.00	0.22	−0.04	0.87	−0.16	0.55
	Male	0.00		0.00		0.00		0.00	
Race	Other Racial Groups	−0.37	0.15	−0.35	0.78	0.41	0.30	0.45	0.27
	White	0.00		0.00		0.00		0.00	
Ethnicity	Hispanic or Latinx	−0.59	0.08	−0.50	0.76	−0.94	0.08	−1.14	0.03
	Not Hispanic or Latinx	0.00		0.00		0.00		0.00	
Highest Academic Degree	Some College/Associate’s Degree	0.25	0.46	3.81	0.02	1.30	0.01	1.28	0.02
	Bachelor’s Degree	0.63	0.05	4.51	<0.01	1.29	0.01	1.25	0.01
	Professional/Graduate Degree	1.10	<0.001	3.87	0.01	1.43	<0.01	1.35	<0.01
	High School/GED	0.00		0.00		0.00		0.00	
Site	University of Florida	0.06	0.72	0.44	0.59	0.03	0.90	−0.10	0.70
	University of Arizona	0.00		0.00		0.00		0.00	
Timepoint	COVID-19 T1	−0.47	<0.01	−1.07	0.03	0.52	0.001	0.90	<0.001
	COVID-19 T2	−0.19	0.24	−0.69	0.13	0.62	<0.001	0.97	<0.001
	COVID-19 T3	−0.17	0.30	−0.81	0.08	0.82	<0.001	1.13	<0.001
	COVID-19 T4	0.02	0.92	−0.26	0.56	0.69	<0.001	1.38	<0.001
	COVID-19 T5	0.22	0.16	−0.21	0.63	1.03	<0.001	1.67	<0.001
	COVID-19 T6	0.39	0.02	−0.88	0.05	1.29	<0.001	1.42	<0.001
	COVID-19 T7	0.39	0.01	−0.74	0.11	1.16	<0.001	1.55	<0.001
	COVID-19 T8	0.52	<0.001	−1.00	0.05	1.13	<0.001	1.57	<0.001
	Pre-Pandemic Baseline	0.00		0.00		0.00		0.00	
Contrast	Pre-Pandemic Baseline vs. COVID-19 T1–T8	0.09	0.46	−0.71	0.08	0.91	<0.001	1.33	<0.001
	COVID-19 T1 vs. COVID-19 T8	0.99	<0.001	0.07	0.87	0.62	<0.001	0.67	<0.001

Notes: PROMIS, Patient-Reported Outcomes Measurement Information System; MoCA 22-Point, Montreal Cognitive Assessment abbreviated version based on 22 points; NACC UDS, National Alzheimer’s Coordinating Center Uniform Data Set. This table contains the model estimates and respective significance values for the main effects of each covariate and timepoint, as well as, the contrasts of interest. The contrast “Pre-Pandemic Baseline vs. COVID-19 T1–T8” compares each measure obtained at a baseline assessment prior to the pandemic to an average of the same measure collected across eight assessments during the pandemic (i.e., the average of COVID-19 timepoints 1 through 8). The contrast “COVID-19 T1 vs. COVID-19 T8” compares each measure obtained at the first assessment during the pandemic compared to the last assessment during the pandemic (i.e., COVID-19 timepoint 1 vs. COVID-19 timepoint 8).

## Results

### Health behaviors

#### AUDIT

**Figure 1:**
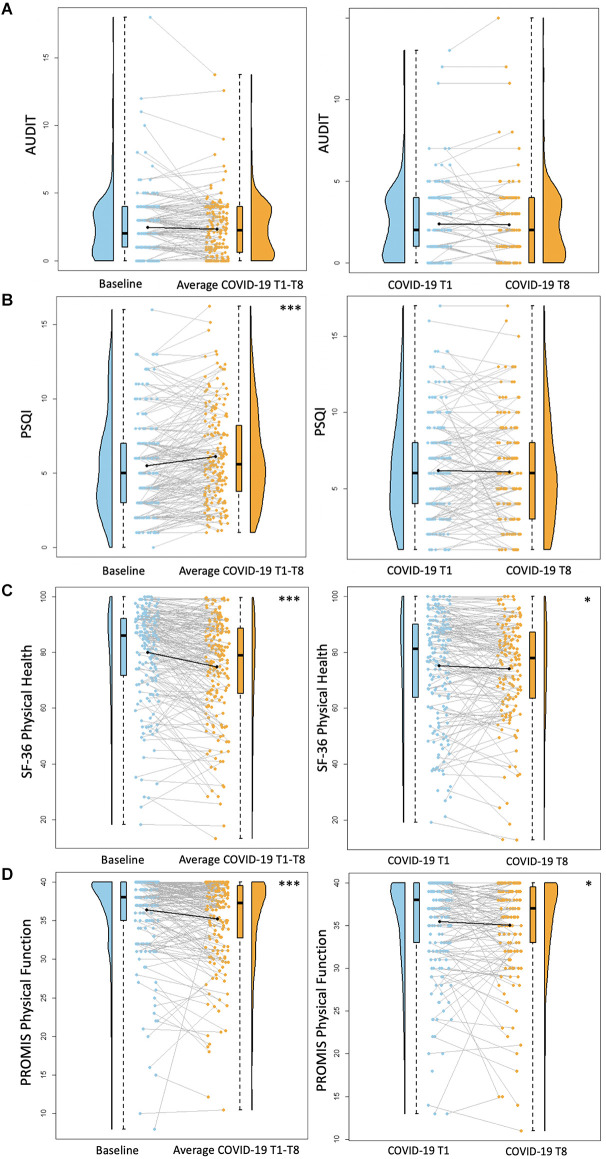
Health behavior results. Plots depicting the raw distribution of data points collected for **(A)** Alcohol Use Disorder Identification Test (AUDIT), **(B)** Pittsburgh Sleep Quality Index (PSQI), **(C)** Short Form 36 Health Survey Questionnaire (SF-36)—Physical Health Summary, and **(D)** Patient-Reported Outcomes Measurement Information System (PROMIS)—Physical Function. The plots on the left side of the panel represent change from pre-pandemic baseline to during the pandemic (average of COVID-19 timepoints 1–8), while the plots on the right side of the panel represent change throughout the pandemic (COVID-19 timepoint 1 vs. timepoint 8). *p < 0.05, ***p < 0.001.

Regarding alcohol use, there were no significant differences between baseline AUDIT scores and average COVID-19 T1–T8 AUDIT scores (*B* = −0.15, *p* = 0.08). There were also no significant differences between COVID-19 T1 AUDIT and COVID-19 T8 AUDIT (*B* = −0.10, *p* = 0.20; Figure 1). Overall, individuals with a professional/graduate degree use more alcohol compared to those with a high school diploma/GED (*B* = 1.99, *p* < 0.001). Individuals from other racial backgrounds use less alcohol compared to White individuals (*B* = −0.98, *p* = 0.04).

#### PSQI

There was a significant increase between baseline PSQI scores and average COVID-19 T1–T8 PSQI scores, suggesting reduced sleep quality during the pandemic compared to pre-pandemic (*B* = 0.63, *p* < 0.001). There were no significant differences between COVID-19 T1 PSQI and COVID-19 T8 PSQI (*B* = 0.04, *p* = 0.82; Figure 1). Females reported worse sleep quality than males (*B* = 1.16, *p* = 0.01).

#### SF-36 Physical Health

There was a significant decrease between baseline SF-36 Physical Health scores and average COVID-19 T1–T8 SF-36 Physical Health scores, suggesting reduced perceived physical health during the pandemic compared to pre-pandemic (*B* = −5.38, *p* < 0.001). Additionally, self-reported physical health was significantly worse at COVID-19 T8 compared to COVID-19 T1 (*B* = −2.19, *p* = 0.02; Figure 1). Overall, older age was associated with worse perceived physical health (*B* = −0.56, *p* = 0.02). Additionally, individuals with higher academic degrees reported better physical health compared to those with a high school diploma/GED (Some college/associate’s degree: *B* = 12.45, *p* = 0.01, bachelor’s degree: *B* = 16.69, *p* < 0.001, professional/graduate degree: *B* = 15.04, *p* = 0.001). Participants recruited from the University of Florida reported worse physical health than those recruited from the University of Arizona (*B* = −6.67, *p* = 0.005).

#### PROMIS Physical Function

There was a significant decrease between baseline PROMIS Physical Function scores and average COVID-19 T1–T8 PROMIS Physical Function scores (*B* = −1.18, *p* < 0.001), suggesting reduced physical function capabilities during the pandemic compared to pre-pandemic. Additionally, self-reported physical function was significantly worse at COVID-19 T8 compared to COVID-19 T1 (*B* = −0.83, *p* = 0.01; Figure 1). Overall, females reported worse physical function compared to males (*B* = −2.04, *p* = 0.01). Individuals with higher academic degrees reported better physical function compared to those with a high school diploma/GED (Some college/associate’s degree: *B* = 3.83, *p* = 0.01, bachelor’s degree: *B* = 4.69, *p* = 0.001, professional/graduate degree: *B* = 4.81, *p* < 0.001). Participants recruited from the University of Florida reported worse physical function than those recruited from the University of Arizona (*B* = −2.02, *p* = 0.01).

### Psychosocial factors

#### SF-36 Mental Health

**Figure 2:**
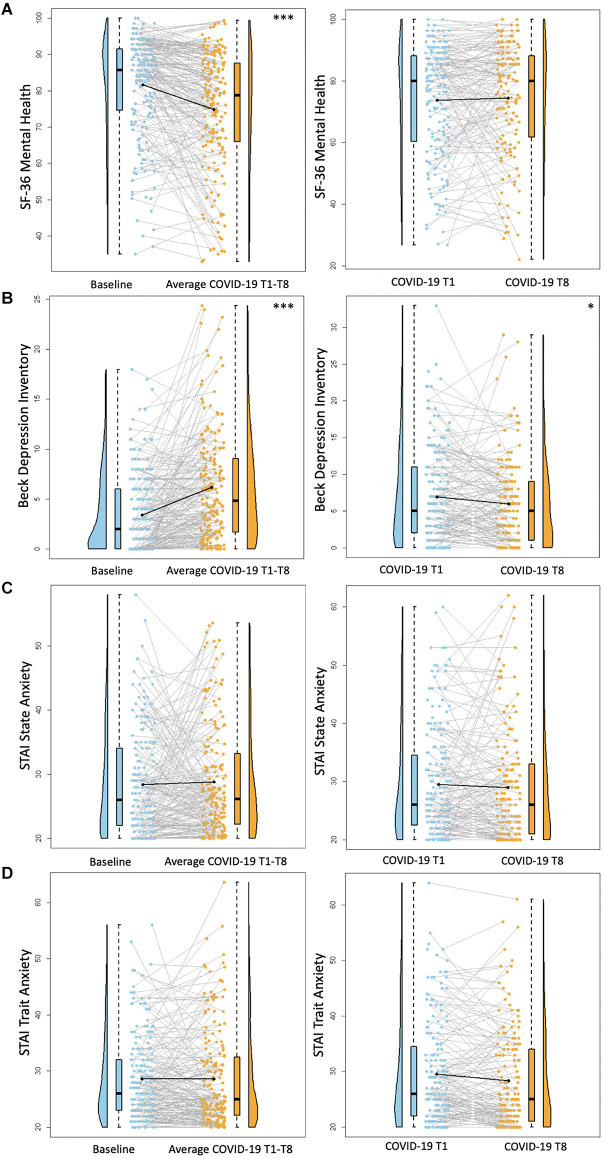
Psychosocial results. Plots depicting the raw distribution of data points collected for **(A)** Short Form 36 Health Survey Questionnaire (SF-36)—Mental Health Summary, **(B)** Beck Depression Inventory—Second Edition, **(C)** State-Trait Anxiety Inventory (STAI)—State Anxiety, and **(D)** State-Trait Anxiety Inventory—Trait Anxiety. The plots on the left side of the panel represent change from pre-pandemic baseline to during the pandemic (average of COVID-19 timepoints 1–8), while the plots on the right side of the panel represent change throughout the pandemic (COVID-19 timepoint 1 vs. timepoint 8). *p < 0.05, ***p < 0.001.

There was a significant decrease between baseline SF-36 Mental Health scores and average COVID-19 T1–T8 SF-36 Mental Health scores (*B* = −7.0, *p* < 0.0001), suggesting poorer self-reported mental health during the pandemic compared to pre-pandemic. There were no significant differences between COVID-19 T1 SF-36 Mental Health and COVID-19 T8 SF-36 Mental Health (*B* = 0.16, *p* = 0.90; Figure 2). Overall, individuals with higher academic degrees reported better mental health compared to those with a high school diploma/GED (Some college/associate’s degree: *B* = 14.22, *p* = 0.0005, bachelor’s degree: *B* = 15.71, *p* = 0.0001, professional/graduate degree: *B* = 15.07, *p* = 0.001).

#### BDI-II

There was a significant increase between baseline BDI-II scores and average COVID-19 T1–T8 BDI-II scores (*B* = 2.75, *p* < 0.001), suggesting greater self-reported depression symptoms during COVID-19 compared to pre-pandemic. Notably, there were significantly lower BDI-II scores at COVID-19 T8 compared to COVID-19 T1 (*B* = −0.89, *p* = 0.04; Figure 2), suggesting an improvement in depression symptoms throughout the pandemic. Overall, older age was associated with reporting greater BDI-II scores (*B* = 0.15, *p* = 0.01). Additionally, individuals with a bachelor’s degree or a professional/graduate degree reported fewer depression symptoms compared to those with a high school diploma/GED (bachelor’s degree: *B* = −2.50, *p* = 0.03, professional/graduate degree: *B* = −2.23, *p* = 0.04). See Table 6 for percentages of the sample delineated by clinical cutoff scores at each timepoint of interest.

**Table 6:** Clinical categorizations for depression, anxiety, and apathy symptoms over time.

	**BDI-II**				**STAI**		**Apathy Scale**	**Total**
**Timepoint**	**Minimal (0–13)**	**Mild (14–19)**	**Moderate (20–28)**	**Severe (29–63)**	**State Anxiety >40**	**Trait Anxiety >40**	**Total >14**	**N**
Pre-Pandemic Baseline	184 (97.4%)	5 (2.6%)	0 (0%)	0 (0%)	23 (12.2%)	19 (10.1%)	26 (13.9%)	189
COVID-19 T1	157 (83.5%)	22 (11.7%)	8 (4.3%)	1 (0.5%)	31 (16.5%)	32 (17.0%)	54 (28.7%)	188
COVID-19 T8	140 (89.7%)	12 (7.7%)	3 (1.9%)	1 (0.6%)	22 (14.1%)	19 (12.2%)	41 (27.7%)	156
COVID-19 T1–T8	167 (88.8%)	13 (6.9%)	8 (4.35%)	0 (0%)	25 (13.2%)	27 (14.4%)	48 (25.5%)	188

Notes: BDI-II, Beck Depression Inventory—Second Edition; STAI, State-Trait Anxiety Inventory.

#### STAI

For both STAI State and STAI Trait anxiety assessments, there were no significant differences between baseline STAI scores and average COVID-19 T1–T8 STAI scores (STAI State *B* = 0.41, *p* = 0.58, STAI Trait *B* = 0.05, *p* = 0.93). Similarly, there were no significant differences between COVID-19 T1 STAI and COVID-19 T8 STAI (STAI State *B* = −0.19, *p* = 0.77, STAI Trait *B* = −0.86, *p* = 0.06; Figure 2). Individuals with some college/associate’s degree or a professional/graduate degree had lower trait anxiety scores compared to those with a high school diploma/GED (Some college/associate’s degree: *B* = −4.79, *p* = 0.02, professional/graduate degree: *B* = −5.52, *p* = 0.01). See Table 6 for percentages of the sample delineated by clinical cutoff scores at each timepoint of interest.

#### Apathy Scale

**Figure 3:**
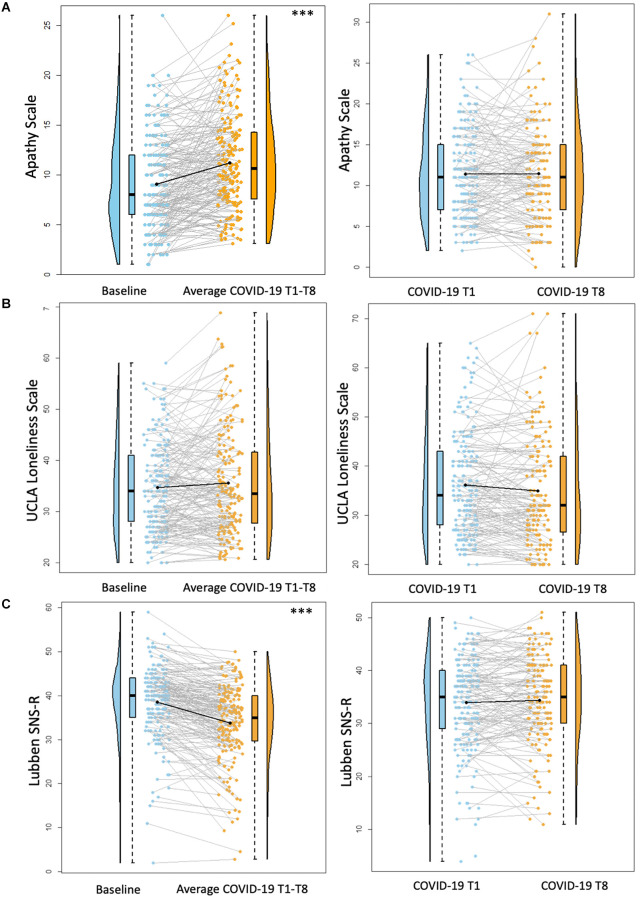
Psychosocial results pt. 2. Plots depicting the raw distribution of data points collected for **(A)** Apathy Scale; **(B)** University of California Los Angeles (UCLA) Loneliness Scale; and **(C)** Lubben Social Network Scale—Revised (SNS-R). The plots on the left side of the panel represent change from pre-pandemic baseline to during the pandemic (average of COVID-19 timepoints 1–8), while the plots on the right side of the panel represent change throughout the pandemic (COVID-19 timepoint 1 vs. timepoint 8). ***p < 0.001.

There was a significant increase between baseline Apathy Scale scores and average COVID-19 T1–T8 Apathy Scale scores (*B* = 2.19, *p* < 0.001), suggesting increased self-reported apathy symptoms during the pandemic compared to pre-pandemic. There were no significant differences between COVID-19 T1 Apathy Scale and COVID-19 T8 Apathy Scale (*B* = 0.25, *p* = 0.48; Figure 3). Individuals with some college/associate’s degree or a professional/graduate degree reported fewer apathy symptoms compared to those with a high school diploma/GED (Some college/associate’s degree: *B* = −2.85, *p* = 0.03, professional/graduate degree: *B* = −3.00, *p* = 0.01). See Table 6 for percentages of the sample delineated by clinical cutoff scores at each timepoint of interest.

#### UCLA Loneliness Scale

There were no significant differences between baseline UCLA Loneliness Scale scores and average COVID-19 T1–T8 Loneliness scores (*B* = 0.95, *p* = 0.09). Additionally, there were no significant differences between COVID-19 T1 UCLA Loneliness Scale and COVID-19 T8 UCLA Loneliness Scale (*B* = −0.52, *p* = 0.36; Figure 3). Overall, individuals with higher academic degrees reported less loneliness compared to individuals with a high school diploma/GED (Some college/associate’s degree: *B* = −6.28, *p* = 0.02, bachelor’s degree: *B* = −5.13, *p* = 0.04, professional/graduate degree: *B* = −6.50, *p* = 0.01).

#### Lubben SNS-R

There was a significant decrease between baseline SNS-R scores and average COVID-19 T1–T8 SNS-R scores (*B* = −4.76, *p* < 0.001), suggesting reduced social engagement/perceived social support during the pandemic compared to pre-pandemic. There were no significant differences between COVID-19 T1 SNS-R and COVID-19 T8 SNS-R (*B* = −0.39, *p* = 0.34; Figure 3). Overall, older age was associated with less reported social engagement/perceived social support (*B* = −0.27, *p* = 0.02). Additionally, individuals with some college/associate’s degree or a professional/graduate degree had better social engagement/perceived social support compared to those with a high school diploma/GED (Some college/Associate’s degree: *B* = 6.67, *p* < 0.01, Professional/Graduate degree: *B* = 4.81, *p* = 0.03).

### Cognitive functioning

#### MoCA

**Figure 4:**
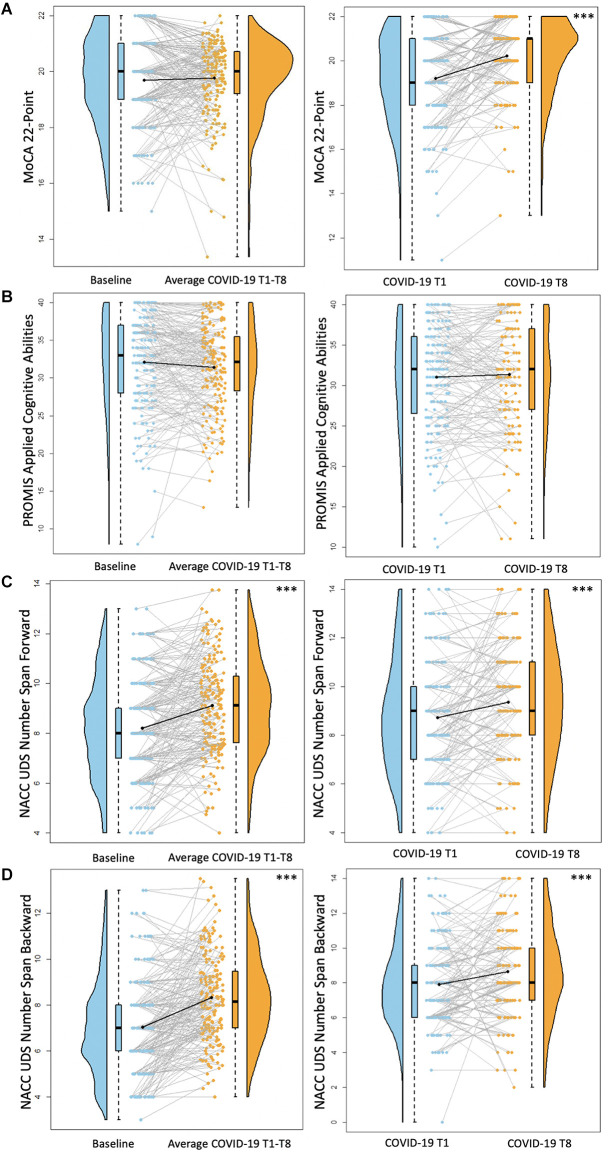
Cognitive functioning results. Plots depicting the raw distribution of data points collected for **(A)** Montreal Cognitive Assessment (MoCA) 22-point version, **(B)** Patient-Reported Outcomes Measurement Information System (PROMIS)–Applied Cognitive Abilities, **(C)** National Alzheimer’s Coordinating Center Uniform Data Set (NACC UDS) Number Span Forward, and **(D)** NACC UDS Number Span Backward. The plots on the left side of the panel represent change from pre-pandemic baseline to during the pandemic (average of COVID-19 timepoints 1–8), while the plots on the right side of the panel represent change throughout the pandemic (COVID-19 timepoint 1 vs. timepoint 8). ***p < 0.001.

For these analyses, the pre-pandemic MoCA score (obtained from the original 30-point scale) was converted to the MoCA 22-point version used for the COVID-19 assessments. There were no differences between screening MoCA scores and average COVID-19 T1–T8 MoCA scores (*B* = 0.09, *p* = 0.46). MoCA scores were significantly better at COVID-19 T8 compared to COVID-19 T1 (*B* = 0.99, *p* < 0.001; Figure 4). Overall, older age was associated with worse MoCA scores (*B* = −0.07, *p* < 0.001). Additionally, individuals with a professional/graduate degree have higher MoCA scores compared to those with a high school diploma/GED (*B* = 1.10, *p* < 0.001).

#### PROMIS Applied Cognition Abilities

There were no significant differences between baseline PROMIS Applied Cognition Abilities scores and average COVID-19 T1–T8 PROMIS Applied Cognition Abilities scores (*B* = −0.71, *p* = 0.08). Additionally, there were no significant differences between COVID-19 T1 PROMIS Applied Cognition Abilities and COVID-19 T8 PROMIS Applied Cognition Abilities (*B* = 0.07, *p* = 0.87; Figure 4). Overall, individuals with higher academic degrees reported better cognitive functioning compared to those with a high school diploma/GED (Some college/associate’s degree: *B* = 3.81, *p* = 0.02, bachelor’s degree: *B* = 4.51, *p* < 0.01, professional/graduate degree: *B* = 3.87, *p* = 0.01).

#### NACC UDS Number Span Test

For both Number Span Forward and Backward trials, there was a significant increase in performance between baseline Number Span scores and average COVID-19 T1–T8 Number scores (Number Span Forward *B* = 0.91, *p* < 0.001, Number Span Backward *B* = 1.33, *p* < 0.001). Additionally, Number Span performance was significantly better at COVID-19 T8 compared to COVID-19 T1 (Number Span Forward *B* = 0.62, *p* < 0.001, Number Span Backward *B* = 0.67, *p* < 0.001; Figure 4). Overall, older age was associated with worse Number Span Forward scores (*B* = −0.05, *p* = 0.04). Hispanic or Latinx individuals performed worse on Number Span Backward compared to Non-Hispanic or Latinx individuals (*B* = −1.14, *p* = 0.03) with a similar ethnicity group difference trending towards statistical significance for Number Span Forward (*B* = −0.94, *p* = 0.08). Further, individuals with higher academic degrees performed better on both tasks compared to those with a high school diploma/GED (Number Span Forward = Some college/associate’s degree: *B* = 1.30, *p* = 0.01, bachelor’s degree: *B* = 1.29, *p* = 0.01, professional/graduate degree: *B* = 1.43, *p* < 0.01; Number Span Backward = Some college/associate’s degree: *B* = 1.28, *p* = 0.02, bachelor’s degree: *B* = 1.25, *p* = 0.01, professional/graduate degree: *B* = 1.35, *p* < 0.01).

## Discussion

The present study evaluated changes in health behaviors, psychosocial factors, and cognitive functioning in a sample of older adults in the context of the COVID-19 pandemic. A particular strength of this study is that it included a pre-pandemic baseline and several follow-up timepoints throughout the pandemic. This allowed for: (1) a comparison of pre-pandemic functioning to an aggregate measure of functioning during the pandemic; and (2) a comparison of functioning between the first and last COVID-19 timepoints to evaluate change throughout the pandemic.

### Health behaviors

This sample of older adults reported worsened sleep quality, perceived physical health, and physical functioning during the pandemic compared to their pre-pandemic baseline. Though the identified health behavior changes were small, these findings are largely consistent with our hypotheses and the previous literature. For example, Almondes et al. (2022) demonstrated that older adults maintained consistent sleep schedules before and during the pandemic; however, they reported slight negative changes in sleep latency, quality, awakenings, and efficiency. Significant stress, anxiety, and depression are all factors that have been associated with poorer sleep quality for individuals during lockdown periods (Franceschini et al., 2020; Xiao et al., 2020). In our sample of older adults, participants did report decreases in mental health during the pandemic compared to baseline; however, there were few individuals that reported clinically significant levels of psychological symptoms. Further, psychological functioning remained largely stable throughout the course of the pandemic. These subclinical changes in mental health findings may explain the small magnitude of change in sleep quality in this healthy older adult population during the pandemic. It would be important to further characterize the pandemic’s effect on sleep within older adult populations that have mood and sleep disorders at baseline.

Older adults have reported reduced engagement in physical activities during the pandemic (Constant et al., 2020; Emerson, 2020; Suzuki et al., 2020). Our study expanded upon these previous findings by assessing older adults’ *perceptions* of general physical health and physical functioning capabilities (i.e., how is your health, and how much does your health limit your ability to complete X task?). In the present sample of older adults, participants reported worsened perceptions of physical health and physical functioning capabilities during the pandemic compared to baseline, which continued to minimally worsen throughout the pandemic (i.e., ~1–2 points on the scales). Importantly, previous studies in older adults have demonstrated that positive expectations and higher self-perception of physical capabilities is associated with increased planning and engagement in physical activity (Breda and Watts, 2017; Sales et al., 2017). As such, the worsening of physical health perceptions in our sample may be a result of their experience with increased sedentary behaviors and systematically reduced engagement in physical activities due to stay-at-home safety procedures. This may be particularly applicable to older adults who rely on access to facilities outside the home (e.g., gyms, physical therapy appointments, community parks) and peer group interactions for exercise (e.g., walking with friends, group classes). Additionally, individuals living in Florida reported worse perceived physical health and functioning compared to those living in Arizona overall, which may be related to a variety of factors like regional differences in health status, in receiving education about exercise, or in access to resources for health maintenance (Kachan et al., 2014; CDC, 2022b). Empowering older adults through providing innovative solutions to engage in physical activity at home during COVID-19 (e.g., gardening, household chores, dancing, online exercise class) may increase physical activity, improve perceptions of physical health and functioning, and also have lasting benefits for preventing cognitive decline (Larson et al., 2006; Hamer et al., 2018; Quigley et al., 2020).

Finally, the present sample of older adults did not experience significant changes in alcohol use compared to baseline or throughout the pandemic. Consistent with previous studies, White older adults reported more alcohol use compared to individuals from other racial backgrounds (Bryant and Kim, 2012, 2019). Alcohol consumption can operate as a coping strategy for alleviating negative emotions and managing stress (Cooper et al., 1992, 1995; Cox and Klinger, 2011). The COVID-19 pandemic presents unique stressors for older adult populations who are at elevated risk for experiencing severe illness from the disease, warranting the investigation of the pandemic’s influence on alcohol consumption. Studies have revealed that older adults report increased consumption of alcohol during the pandemic; however, increases were greater in younger adults (Niedzwiedz et al., 2021; Capasso et al., 2021). This pattern aligns with theories that suggest older adults use effective coping strategies to regulate stress more frequently than younger adults due to an awareness of diminished temporal horizons and accrual of expertise from life experiences (Carstensen et al., 2003; Charles, 2010). Indeed, older adults endorsed more proactive coping than younger adults during the pandemic, which was associated with less pandemic-related stress (Pearman et al., 2021). Furthermore, increased alcohol consumption during the pandemic in older adults was greater among those with mental health symptomatology (Capasso et al., 2021; Eastman et al., 2021). Taken together, the stability of alcohol consumption in our sample may be reflective of: (1) successful employment of adaptive coping strategies to navigate pandemic-related stress; and/or (2) a relatively low impact of these stressors on emotional functioning forgoing the need to engage in coping strategies given the reports of largely subclinical mood symptoms.

### Psychosocial factors

This sample of older adults had lower scores on a mental health composite and reported more depression and apathy symptoms during the pandemic compared to their pre-pandemic baseline. Throughout the pandemic, measures of psychological functioning remained stable except that participants reported fewer depression symptoms over time. There were no significant changes in anxiety relative to baseline or throughout the pandemic. Prior findings regarding the impact of COVID-19 on mental health outcomes in older adults has been heterogenous, revealing both no changes in psychological functioning (Hamm et al., 2020; Pierce et al., 2020; van der Velden et al., 2020; Daly et al., 2021; Kivi et al., 2021) and negative changes (Shan Wong et al., 2020; Krendl and Perry, 2021). The observed changes in self-reported depression and apathy symptoms in our sample should be interpreted with caution, considering the difference between statistical significance and real-world clinical significance. For instance, on average, depression and apathy total scores increased about 3 and 2 points respectively relative to a pre-pandemic baseline. Further, depression scores decreased about 1 point over the course of the pandemic. These changes are not large enough to affect the clinical categorization of symptom severity for several individuals except for those on categorical boundaries. Overall, this sample largely remained below clinically elevated cutoffs for depression and apathy across timepoints. For instance, at baseline 97.4% reported minimal depression symptoms while 2.6% reported mild depression symptoms. Throughout the pandemic, 88.8% reported minimal depression symptoms, 6.9% reported mild depression symptoms, and 4.3% reported moderate depression symptoms. Similarly, at baseline, 13.9% were above the clinical cutoff for apathy symptoms, while 25.5% were above the cutoff during the pandemic. Therefore, it is important to not over-extrapolate these findings by suggesting a clinically negative impact of the pandemic on self-reported mental health outcomes in this sample of older adults. Notably, the parent trial initially excluded individuals reporting moderate to severe depression symptoms. As such, the current findings may not generalize to older adults with clinically elevated depression or psychological symptoms, and future research should examine the longitudinal impact of the pandemic in these populations.

Notably, there may be age-related differences in the pandemic’s impact on psychological functioning. Compared to younger adults, older adults have reported greater emotional well-being and higher resilience during the pandemic (Carstensen et al., 2020; Carbone et al., 2021; Ceccato et al., 2021). This identified pattern and the current findings seem to align with the socioemotional selectivity theory (Carstensen et al., 2003), which posits that older adults experience motivational shifts towards achieving emotional meaning and psychological well-being, as future time is perceived as constrained. As such, older adults may experience an advantage coping with the emotional and psychological sequelae of the COVID-19 pandemic. Future work should identify factors to explain individual variability in the pandemic’s impact on psychological functioning to better identify those at risk for experiencing clinically significant changes and develop intervention strategies to support these individuals.

Regarding social isolation and loneliness, this sample of older adults reported less social engagement/perceived social support but no changes in loneliness during the pandemic compared to their pre-pandemic baseline. Social isolation and loneliness are independent constructs, as the former refers to the objective lack of social connections and relationships, while the latter refers to a subjective feeling of isolation regardless of the number of social contacts (Coyle and Dugan, 2012). In older adults, loneliness has only been modestly correlated with measures of social isolation and network size (Routasalo et al., 2006; Coyle and Dugan, 2012) but is more so related to expectations and satisfaction with social contacts (Routasalo et al., 2006). A recent meta-analysis across 34 eligible studies in over 200,000 participants demonstrated an increase in loneliness during COVID-19 relative to pre-pandemic timepoints; however, the effects were small and heterogenous (Ernst et al., 2022). In our sample, the reported reduction in social engagement can be expected given the implementation of social distancing policies for health safety purposes during the pandemic, but perhaps, the quality of these connections was maintained and protected against feelings of loneliness. This speculation also aligns with the socioemotional selectivity theory, suggesting that older adults’ may be better able to optimize fewer social connections without experiencing loneliness (Carstensen et al., 2003). Future research should particularly examine factors like quality of or satisfaction with social networks, living status (e.g., alone vs. with others), access to internet, and use of technology for communication with others to identify populations at risk for potentially experiencing social isolation and loneliness during the pandemic.

### Cognitive functioning

On average, this sample of older adults scored about 1–1.3 points higher on an attention and a working memory task (i.e., NACC UDS Number Span Forward and Backward, respectively) during the pandemic compared to a pre-pandemic baseline. Throughout the pandemic, participants demonstrated a slight improvement on both of these tasks (~0.6–0.7 points), which is generally consistent with the magnitude of reported performance gains after repeated administration of a similar task over a short period of time (i.e., Digit Span; Wilson et al., 2000; Coalson and Raiford, 2008; Estevis et al., 2012). Additionally, throughout the pandemic, participants’ total score on a general cognitive screener (i.e., MoCA) increased on average by one point despite alternating between three versions of the test. However, the magnitude of change is consistent with the MoCA validation study (Nasreddine et al., 2005), which demonstrated a 0.9 ± 2.5-point mean change in MoCA scores between two evaluations administered about 35 days apart. There were also no significant changes on a subjective measure of cognitive abilities.

Overall, both subjective and objective cognitive functioning remained largely stable or slightly improved across this sample of healthy older adults in the context of the COVID-19 pandemic. This pattern aligns with the few previous studies demonstrating stability in subjective cognitive complaints, general cognition, and working memory performance in older adults during the pandemic (Amanzio et al., 2021; Carbone et al., 2021; De Pue et al., 2021). Furthermore, aside from potential practice effects, the slight improvements in cognitive functioning may reflect benefits from socialization received through study visits and research staff interaction. For instance, using an ecological approach and monitoring outcomes in real time, Zhaoyang et al. (2021) demonstrated fluctuations in older adults’ cognitive functioning, such that those with more frequent daily social interactions have better cognitive performance on the same day and over the following 2 days. While the current sample reported less social engagement during the pandemic compared to baseline on average, perhaps the frequent study visits and phone calls following their baseline assessment and throughout the pandemic facilitated momentary improvements in cognitive functioning captured on assessment. Future research should include a more extensive neurocognitive battery and assess the pandemic’s impact on cognitive changes in older adults at a greater risk for progressing to dementia (i.e., those with mild cognitive impairment).

### Demographics differences

The present study also revealed notable demographic differences in reports of health behaviors, psychosocial factors, and cognitive functioning. In our sample, older age was significantly associated with worse perceived physical health, greater self-reported depression symptoms, less social engagement/perceived social support, and worse general cognition and attention scores. These factors are significantly interconnected and are intervention targets for optimizing functioning in aging populations (Santos et al., 2014; Yates et al., 2017; Lazar et al., 2021). Our findings further highlight the importance of recommending “brain health” interventions (i.e., maintaining mental well-being, social connectedness, exercise, cognitive stimulation; Mintzer et al., 2019) particularly for older individuals within the 65–89 age bracket. Additionally, in our sample, Hispanic or Latinx individuals had lower scores than non-Hispanic or Latinx individuals on the Number Span Backward test. This pattern is consistent with a prior study examining racial/ethnic differences in NACC UDS subtest performance, and controlling for these baseline differences in longitudinal models eliminated racial/ethnic differences in NACC UDS subtest performance change over time (Chuen et al., 2021). It will be important for future research to further identify contributing factors to facilitate a more comprehensive interpretation of racial/ethnic differences on NACC UDS performance.

There was also a main effect of sex on sleep quality, such that females reported worse sleep quality than males overall. This pattern may be explained by various factors including predisposed increased risk for sleep disturbance in older adult females (Zhang and Wing, 2006) or worse general mental health in late life for females compared to males (Sialino et al., 2020), which can negatively affect sleep behaviors (Magee and Carmin, 2010; Becker et al., 2016; Weeks et al., 2019). While the current study did not identify statistically significant sex-differences in reported depression, anxiety, or apathy symptoms, females did report lower scores on an overall mental health composite compared to males, which trended towards significance (*p* = 0.08). As such, there may be subtle sex-differences in the psychological functioning of this healthy older adult sample that may in part explain sex-differences in sleep quality.

Finally, education largely served as a protective factor, as older adults with higher levels of education reported better physical health, physical functioning, general mental health, social engagement/perceived support, subjective and objective cognitive functioning, less loneliness, had lower trait anxiety, and reported fewer depression and apathy symptoms compared to those with a high school diploma or GED. Educational attainment is one of several social determinants of health, well-being, and longevity driven by systemic racism and mediated through economic advantages, social-psychological factors, and access to health care resources (Maness et al., 2021; see Zajacova and Lawrence, 2018 for review). Indeed, throughout the COVID-19 pandemic, there is further evidence of this disparity as lower educational attainment has been associated with increased risk of COVID-19 infection and hospitalization (Chadeau-Hyam et al., 2020; Li et al., 2021). These findings have important implications regarding the need for dismantling educational barriers, especially as the COVID-19 pandemic has further widened pre-existing disparities in educational opportunities, particularly for Black, Latinx, and American Indian or Alaska Native students, with the transition of in-person schooling to online learning (Office of Civil Rights, 2021).

## Limitations

While the present study provides longitudinal assessments prior to and during the COVID-19 pandemic across health behavior, psychosocial factors, and cognitive domains in older adults, the results must be considered with the following limitations. As previously stated, some of the statistically significant changes were rather small in terms of clinical significance with only a few point differences on average between assessments. Therefore, it is important to not overinterpret the impact of the pandemic on these outcomes. Although the sample as a whole did not drastically experience changes compared to their baseline or throughout the pandemic, there is likely individual variability in the magnitude of these changes. However, the ability to examine this variability in the present study is in part limited by the lack of racial and ethnic representation in our sample. The sample consisted primarily of White and non-Hispanic individuals, which generally thwarted the ability to detect differences between racial and ethnic groups in outcomes. Compared to non-Hispanic White individuals, American Indian or Alaska Native, Black or African American, and Hispanic individuals are more likely to contract, be hospitalized, and die from COVID-19 (CDC, 2022a). A recent study used the World Health Organization’s Health Inequity Causal Model and identified factors driving these racial and ethnic disparities among older adults for COVID-19 hospitalizations and deaths including systemic racism and the resulting economic context, exposure risk due to living arrangements (i.e., densely populated settings, multi-generational households), increased vulnerability due to chronic conditions, and experiencing severe stages of the disease due to health system barriers (Guerrero and Wallace, 2021). This further aligns with the National Institute on Aging’s framework to include environmental, sociocultural, behavioral, and biological factors to better identify causal pathways in health disparity research, which would be important for future pandemic-related studies (Hill et al., 2015). The present study does not capture the disproportionately adverse effects of the pandemic experienced by these individuals. There may have been selection bias in recruitment strategies that contributed to the lack of racial and ethnic representation in the sample. Furthermore, participants who experienced greater negative impacts of the pandemic may have been less willing to continue to participate in the research study throughout COVID-19. Taken together, future research should replicate this study design to better examine individual differences in the impact of COVID-19 across health behaviors, psychosocial factors, and cognitive domains in a diverse, representative, and unbiased sample of older adults. If the current patterns of findings are replicated in such a sample, that may further provide support for age-related resilience in response to a global pandemic.

## Conclusions

The present study provides insights into the impact of the COVID-19 pandemic and resulting isolation procedures on health behaviors, psychosocial factors, and cognitive functioning in a population at risk for contracting and experiencing severe illness from the disease. In brief, our sample of older adults reported worsened sleep quality, perceived physical health and functioning, mental health, increased depression and apathy symptoms, and reduced social engagement/perceived social support during the pandemic compared to their pre-baseline, which may have resulted from increased stress, health-related concerns, and isolation policies implemented as a response to the pandemic. In contrast, this sample demonstrated slightly better performance on objective cognitive tasks of attention and working memory during the pandemic compared to their pre-pandemic baseline potentially related to increased socialization through frequent study visits. Throughout the pandemic, these older adults continued to report worsened perceived physical health and function; however, they reported fewer depression symptoms and further demonstrated improved cognitive performance. Worsening perceptions of physical health and function may have been related to prolonged reductions in physical activity engagement and increased sedentary behaviors due to closures of exercise facilities and encouragement of social distancing throughout the early months of the pandemic. While improvements in depression symptoms may reflect the use of advantageous emotional coping strategies in aging (i.e., socioemotional selectivity theory), it is important to note that the change in depression scores was small and likely not clinically meaningful. Similarly, the slight improvements in cognitive performance throughout the pandemic were comparable in size to practice effects previously demonstrated in the literature.

Regarding demographic differences, women reported worse sleep quality compared to men, Florida residents reported worse perceived physical health and functioning compared to Arizona residents, White individuals reported greater alcohol use, and Hispanic or Latinx individuals performed worse on a working memory task. Importantly, older age was negatively associated with perceived physical health, depression symptoms, social engagement/perceived social support, and general cognition and attention performance. Further encouragement of “brain health” interventions in advanced age will be important for optimizing daily functioning in this population. Finally, individuals with higher levels of education reported better perceived physical health, physical functioning, general mental health, social engagement/perceived support, subjective and objective cognitive functioning, and less loneliness, trait anxiety, depression, and apathy symptoms. This finding further supports extensive evidence suggesting educational attainment serves as a protective factor particularly in late life and has important future implications given the disruption in access to education during the pandemic. Overall, the changes in health behaviors, psychosocial factors, and cognitive functioning compared to a pre-pandemic baseline and throughout the first 9 months of the COVID-19 pandemic were statistically significant yet relatively small in size. Replicating this type of design in a large demographically representative sample would further expand our understanding about the impact of the COVID-19 pandemic on a diverse older adult population and inform intervention strategies to best maintain functioning across individuals.

## Data Availability Statement

The raw data supporting the conclusions of this article will be made available by the authors, without undue reservation.

## Ethics Statement

The studies involving human participants were reviewed and approved by Institutional Review Board at the University of Florida and at the University of Arizona. The patients/participants provided their written informed consent to participate in this study.

## Author Contributions

HH and AW contributed to the conception and design of this specific analysis. YD extracted the data and performed the statistical analyses. HH wrote the first draft of the manuscript. EP, GH, SW, SD, GA, MM, RC, and AW were involved in project administration. VD, MF, KC, DC, CH, and SP were involved in project logistics and data collection. All authors contributed to the article and approved the submitted version.

## Funding

This work was supported by the National Institute on Aging [NIA R01AG054077, NIA P30AG019610, T32AG020499], the State of Arizona and Arizona Department of Health Services (ADHS), the University of Florida Center for Cognitive Aging and Memory Clinical Translational Research, and the McKnight Brain Research Foundation.

## Footnotes

^1^ https://clinicaltrials.gov/ct2/show/NCT02851511
